# 2,3-Diamino­pyridinium sorbate–sorbic acid (1/1)

**DOI:** 10.1107/S1600536811053025

**Published:** 2011-12-21

**Authors:** Madhukar Hemamalini, Jia Hao Goh, Hoong-Kun Fun

**Affiliations:** a X-ray Crystallography Unit, School of Physics, Universiti Sains Malaysia, 11800 USM, Penang, Malaysia

## Abstract

In the title mol­ecular salt–adduct, C_5_H_8_N_3_^+^·C_6_H_7_O_2_^−^·C_6_H_8_O_2_, the 2,3-diamino­pyridinium cation is essentially planar, with a maximum deviation of 0.013 (2) Å, and is protanated at its pyridine N atom. The sorbate anion and sorbic acid mol­ecules exist in extended conformations. In the crystal, the protonated N atom and one of the two amino-group H atoms are hydrogen bonded to the sorbate anion through a pair of N—H⋯O hydrogen bonds, forming an *R*_1_^2^(6) ring motif. The carboxyl groups of the sorbic acid mol­ecules and the carboxyl­ate groups of the sorbate anions are connected *via* O—H⋯O hydrogen bonds. Furthermore, the ion pairs and neutral mol­ecules are connected *via* inter­molecular N—H⋯O hydrogen bonds, forming sheets lying parallel to (100).

## Related literature

For a different crystal structure arising from the same synthesis conditions, see: Hemamalini & Fun (2010 ▶). For background to amino­pyridines, see: Peng *et al.* (2001 ▶); Leung *et al.* (2002 ▶); Banerjee & Murugavel (2004 ▶); Lautie & Belabbes (1996 ▶). For hydrogen-bond motifs, see: Bernstein *et al.* (1995 ▶). For the stability of the temperature controller used in the data collection, see: Cosier & Glazer (1986 ▶).
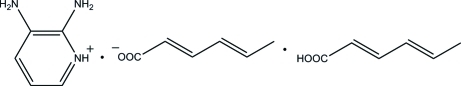


## Experimental

### 

#### Crystal data

C_5_H_8_N_3_^+^·C_6_H_7_O_2_^−^·C_6_H_8_O_2_
*M* *_r_* = 333.38
Monoclinic, 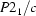

*a* = 16.1636 (17) Å
*b* = 9.6538 (10) Å
*c* = 12.6887 (13) Å
β = 112.844 (2)°
*V* = 1824.6 (3) Å^3^
*Z* = 4
Mo *K*α radiation
μ = 0.09 mm^−1^
*T* = 100 K
0.47 × 0.25 × 0.06 mm

#### Data collection

Bruker APEXII DUO CCD diffractometer
Absorption correction: multi-scan (*SADABS*; Bruker, 2009 ▶) *T*_min_ = 0.960, *T*_max_ = 0.994
27805 measured reflections
5345 independent reflections
2898 reflections with *I* > 2σ(*I*)
*R*_int_ = 0.045

#### Refinement

*R*[*F* ^2^ > 2σ(*F* ^2^)] = 0.047
*wR*(*F* ^2^) = 0.152
*S* = 1.02
5345 reflections
239 parameters
H atoms treated by a mixture of independent and constrained refinement
Δρ_max_ = 0.30 e Å^−3^
Δρ_min_ = −0.22 e Å^−3^

### 

Data collection: *APEX2* (Bruker, 2009 ▶); cell refinement: *SAINT* (Bruker, 2009 ▶); data reduction: *SAINT*; program(s) used to solve structure: *SHELXTL* (Sheldrick, 2008 ▶); program(s) used to refine structure: *SHELXTL*; molecular graphics: *SHELXTL*; software used to prepare material for publication: *SHELXTL*and *PLATON* (Spek, 2009 ▶).

## Supplementary Material

Crystal structure: contains datablock(s) global, I. DOI: 10.1107/S1600536811053025/hb6558sup1.cif

Structure factors: contains datablock(s) I. DOI: 10.1107/S1600536811053025/hb6558Isup2.hkl

Supplementary material file. DOI: 10.1107/S1600536811053025/hb6558Isup3.cml

Additional supplementary materials:  crystallographic information; 3D view; checkCIF report

## Figures and Tables

**Table 1:** Hydrogen-bond geometry (Å, °)

*D*—H⋯*A*	*D*—H	H⋯*A*	*D*⋯*A*	*D*—H⋯*A*
O1*A*—H1*A*⋯O1*B*	0.82	1.79	2.5252 (19)	148
N1—H1*N*1⋯O1*A*^i^	0.89 (3)	2.06 (3)	2.887 (2)	153 (3)
N1—H1*N*1⋯O2*A*^i^	0.89 (3)	2.31 (3)	3.094 (2)	147 (2)
N2—H1*N*2⋯O1*A*^i^	0.83 (2)	2.30 (2)	3.054 (2)	152 (2)
N2—H1*N*2⋯O2*B*^i^	0.83 (2)	2.59 (3)	3.136 (2)	125 (2)
N2—H2*N*2⋯O2*A*	0.86 (2)	2.00 (3)	2.863 (2)	177 (2)
N3—H1*N*3⋯O2*A*	0.84 (2)	2.19 (2)	3.010 (2)	166.1 (16)
N3—H2*N*3⋯O2*B*^ii^	0.92 (3)	2.09 (3)	3.002 (2)	170 (2)

Symmetry codes: (i) 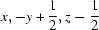; (ii) 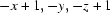.

**Fig. 1.**
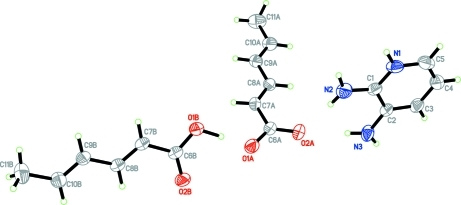
The asymmetric unit of the title compound, showing 50% probability displacement ellipsoids.

**Fig. 2.**
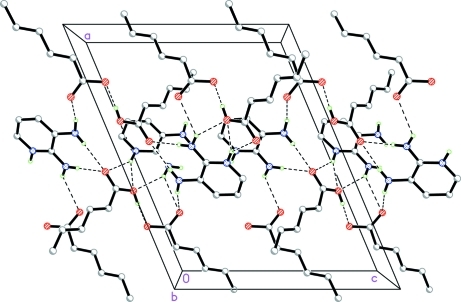
The crystal packing of title compound (I).

## supplementary crystallographic information

### Comment

Aminopyridines have recently become the focus of extensive studies, mainly because of their wide use as building blocks for synthetic transformations (Peng *et al.*, 2001; Leung *et al.*, 2002). Carboxylic acids are important in crystal engineering due to their strong and directional O—H···O and N—H···O hydrogen bonds; this is the main hydrogen-bonding motif often encountered in carboxylic acid–amine complexes (Banerjee & Murugavel, 2004; Lautie & Belabbes, 1996). Here, we report the synthesis and crystal structure of the title compound, (I).

The asymmetric unit of the title compound, (Fig 1), contains one 2,3-diaminopyridinium cation, one sorbate anion and one neutral sorbic acid molecule.  The 2,3-diaminopyridinium cation is planar with a maximum deviation of 0.013 (2) Å for atom C2. Protonation of atom N1 has resulted in a slight increase in the angle C1—N1—C5 [123.71 (17)°]. The sorbate anion and sorbic acid moiety is in the *EE* configuration. The structure is significantly different chemically and structurally from that of the previously reported 2,3-diaminopyridinium (2*E*,4*E*)-hexa-2,4- dienoate compound C_5_H_8_N_3_^+^, C_6_H_7_O_2_^-^ (Hemamalini & Fun, 2010), even though the same synthesis was used.

In the crystal, (Fig. 2), the protonated N1 atom and the 2-amino group N atom (N2) is hydrogen-bonded to the carboxylate oxygen atoms (O1A and O2A) *via* a pair of N—H···O hydrogen bonds forming a ring motif *R*^1^_2_(6) (Bernstein *et al.*, 1995). The carboxyl groups of the sorbic acid molecules and the carboxylate groups of the sorbate anions are connected *via* O—H···O hydrogen bonds.  Furthermore, the ion pairs and neutral molecules are connected *via* N—H···O hydrogen bonds (see Table 1 for symmetry codes) forming two-dimensional networks parallel to (100).

### Experimental

A hot methanol solution (20 ml) of 2,3-diaminopyridine (59 mg, Aldrich) and sorbic acid (56 mg, Merck) were mixed and warmed over a heating magnetic stirrer hotplate for a few minutes. The resulting solution was allowed to cool slowly at room temperature and brown plates of the title compound appeared after a few days.

### Refinement

Atoms H1N1, H1N2, H2N2, H1N3 and H2N3 were located from a difference Fourier maps and refined freely [N–H = 0.83 (2)–0.92 (2) Å]. The remaining H atoms were positioned geometrically [C–H = 0.93–0.96 Å and O–H = 0.82 Å] and were refined using a riding model, with *U*_iso_(H) = 1.2 or 1.5 *U*_eq_(C). A rotating group model was used for the methyl group.

### Crystal data

**Table:**

C_5_H_8_N_3_^+^·C_6_H_7_O_2_^−^·C_6_H_8_O_2_	*F*(000) = 712
*M**_r_* = 333.38	*D*_x_ = 1.214 Mg m^−^^3^
Monoclinic, *P*2_1_/*c*	Mo *K*α radiation, λ = 0.71073 Å
Hall symbol: -P 2ybc	Cell parameters from 4699 reflections
*a* = 16.1636 (17) Å	θ = 2.5–23.6°
*b* = 9.6538 (10) Å	µ = 0.09 mm^−^^1^
*c* = 12.6887 (13) Å	*T* = 100 K
β = 112.844 (2)°	Plate, brown
*V* = 1824.6 (3) Å^3^	0.47 × 0.25 × 0.06 mm
*Z* = 4	

### Data collection

**Table:**

Bruker APEXII DUO CCD diffractometer	5345 independent reflections
Radiation source: fine-focus sealed tube	2898 reflections with *I* > 2σ(*I*)
graphite	*R*_int_ = 0.045
φ and ω scans	θ_max_ = 30.1°, θ_min_ = 2.5°
Absorption correction: multi-scan (*SADABS*; Bruker, 2009)	*h* = −22→22
*T*_min_ = 0.960, *T*_max_ = 0.994	*k* = −13→13
27805 measured reflections	*l* = −17→17

### Refinement

**Table:**

Refinement on *F*^2^	Primary atom site location: structure-invariant direct methods
Least-squares matrix: full	Secondary atom site location: difference Fourier map
*R*[*F*^2^ > 2σ(*F*^2^)] = 0.047	Hydrogen site location: inferred from neighbouring sites
*wR*(*F*^2^) = 0.152	H atoms treated by a mixture of independent and constrained refinement
*S* = 1.02	*w* = 1/[σ^2^(*F*_o_^2^) + (0.0631*P*)^2^ + 0.2243*P*] where *P* = (*F*_o_^2^ + 2*F*_c_^2^)/3
5345 reflections	(Δ/σ)_max_ < 0.001
239 parameters	Δρ_max_ = 0.30 e Å^−^^3^
0 restraints	Δρ_min_ = −0.22 e Å^−^^3^

### Special details

**Table:**

Experimental. The crystal was placed in the cold stream of an Oxford Cryosystems Cobra open-flow nitrogen cryostat (Cosier & Glazer, 1986) operating at 100.0 (1) K.
Geometry. All s.u.'s (except the s.u. in the dihedral angle between two l.s. planes) are estimated using the full covariance matrix. The cell s.u.'s are taken into account individually in the estimation of s.u.'s in distances, angles and torsion angles; correlations between s.u.'s in cell parameters are only used when they are defined by crystal symmetry. An approximate (isotropic) treatment of cell s.u.'s is used for estimating s.u.'s involving l.s. planes.
Refinement. Refinement of F^2^ against ALL reflections. The weighted R-factor wR and goodness of fit S are based on F^2^, conventional R-factors R are based on F, with F set to zero for negative F^2^. The threshold expression of F^2^ > 2σ(F^2^) is used only for calculating R-factors(gt) etc. and is not relevant to the choice of reflections for refinement. R-factors based on F^2^ are statistically about twice as large as those based on F, and R- factors based on ALL data will be even larger.

### Fractional atomic coordinates and isotropic or equivalent isotropic displacement parameters (Å^2^)

**Table:**

	*x*	*y*	*z*	*U*_iso_*/*U*_eq_	
N1	0.52439 (11)	0.20084 (17)	0.03419 (12)	0.0703 (4)	
N2	0.45891 (11)	0.1854 (2)	0.16546 (18)	0.0778 (5)	
N3	0.59685 (10)	0.00896 (17)	0.29774 (14)	0.0713 (4)	
C1	0.52362 (10)	0.14816 (17)	0.13081 (13)	0.0563 (4)	
C2	0.59314 (9)	0.05457 (16)	0.19419 (13)	0.0522 (4)	
C3	0.65504 (10)	0.02052 (18)	0.14930 (15)	0.0642 (4)	
H3A	0.7001	−0.0427	0.1878	0.077*	
C4	0.65219 (12)	0.0785 (2)	0.04688 (16)	0.0771 (5)	
H4A	0.6950	0.0548	0.0179	0.093*	
C5	0.58688 (14)	0.1688 (2)	−0.00887 (16)	0.0808 (5)	
H5A	0.5844	0.2092	−0.0766	0.097*	
O1B	0.20615 (8)	0.22997 (14)	0.43760 (10)	0.0701 (3)	
O2B	0.26440 (7)	0.21636 (13)	0.62722 (10)	0.0698 (3)	
C6B	0.20207 (10)	0.24501 (15)	0.53806 (14)	0.0541 (4)	
C7B	0.11588 (10)	0.29993 (16)	0.53419 (14)	0.0560 (4)	
H7BA	0.0703	0.3165	0.4631	0.067*	
C8B	0.10027 (10)	0.32670 (15)	0.62678 (13)	0.0536 (4)	
H8BA	0.1460	0.3066	0.6969	0.064*	
C9B	0.01845 (10)	0.38450 (16)	0.62879 (14)	0.0569 (4)	
H9BA	−0.0277	0.4038	0.5588	0.068*	
C10B	0.00417 (13)	0.41200 (19)	0.72185 (16)	0.0688 (5)	
H10A	0.0505	0.3925	0.7916	0.083*	
C11B	−0.07969 (15)	0.4715 (3)	0.7250 (2)	0.0929 (7)	
H11A	−0.1059	0.4066	0.7604	0.139*	
H11B	−0.1213	0.4904	0.6485	0.139*	
H11C	−0.0660	0.5560	0.7683	0.139*	
O1A	0.35766 (7)	0.14675 (14)	0.44960 (11)	0.0747 (4)	
H1A	0.3058	0.1418	0.4452	0.112*	
O2A	0.43810 (7)	0.06465 (17)	0.35977 (11)	0.0830 (4)	
C6A	0.36773 (10)	0.06657 (18)	0.37630 (13)	0.0588 (4)	
C7A	0.29051 (10)	−0.02288 (17)	0.31033 (13)	0.0571 (4)	
H7AA	0.2396	−0.0174	0.3273	0.068*	
C8A	0.28871 (10)	−0.10933 (17)	0.22971 (13)	0.0571 (4)	
H8AA	0.3407	−0.1171	0.2153	0.069*	
C9A	0.21322 (11)	−0.19332 (17)	0.16161 (14)	0.0592 (4)	
H9AA	0.1603	−0.1820	0.1733	0.071*	
C10A	0.21248 (13)	−0.28488 (19)	0.08416 (16)	0.0716 (5)	
H10B	0.2656	−0.2947	0.0726	0.086*	
C11A	0.13681 (15)	−0.3735 (2)	0.01381 (18)	0.0891 (6)	
H11G	0.1245	−0.3584	−0.0657	0.134*	
H11D	0.0846	−0.3507	0.0288	0.134*	
H11E	0.1521	−0.4690	0.0325	0.134*	
H1N1	0.4814 (17)	0.260 (3)	−0.006 (2)	0.112 (8)*	
H1N2	0.4194 (16)	0.234 (2)	0.1193 (19)	0.093 (7)*	
H2N2	0.4547 (15)	0.148 (3)	0.225 (2)	0.098 (9)*	
H1N3	0.5524 (13)	0.0094 (18)	0.3168 (15)	0.066 (5)*	
H2N3	0.6417 (15)	−0.055 (3)	0.3295 (19)	0.104 (7)*	

### Atomic displacement parameters (Å^2^)

**Table:**

	*U*^11^	*U*^22^	*U*^33^	*U*^12^	*U*^13^	*U*^23^
N1	0.0674 (9)	0.0770 (10)	0.0511 (8)	0.0190 (8)	0.0062 (7)	0.0022 (7)
N2	0.0622 (9)	0.0905 (12)	0.0739 (11)	0.0316 (9)	0.0190 (8)	−0.0020 (10)
N3	0.0521 (8)	0.0816 (11)	0.0848 (11)	0.0154 (8)	0.0314 (8)	0.0260 (9)
C1	0.0475 (8)	0.0591 (9)	0.0528 (9)	0.0076 (7)	0.0093 (6)	−0.0078 (7)
C2	0.0435 (7)	0.0516 (8)	0.0558 (9)	0.0029 (6)	0.0132 (6)	0.0003 (7)
C3	0.0476 (8)	0.0699 (10)	0.0708 (11)	0.0105 (7)	0.0181 (7)	−0.0003 (9)
C4	0.0663 (11)	0.1024 (15)	0.0656 (11)	0.0092 (10)	0.0288 (9)	−0.0031 (11)
C5	0.0818 (13)	0.1025 (15)	0.0527 (10)	0.0082 (11)	0.0203 (9)	0.0028 (10)
O1B	0.0584 (7)	0.0927 (9)	0.0518 (6)	0.0105 (6)	0.0133 (5)	−0.0043 (6)
O2B	0.0546 (6)	0.0833 (8)	0.0586 (7)	0.0206 (6)	0.0077 (5)	0.0004 (6)
C6B	0.0472 (8)	0.0474 (8)	0.0572 (9)	0.0013 (6)	0.0087 (7)	−0.0020 (7)
C7B	0.0465 (8)	0.0593 (9)	0.0530 (9)	0.0042 (7)	0.0091 (6)	0.0043 (7)
C8B	0.0484 (8)	0.0488 (8)	0.0542 (9)	0.0001 (6)	0.0097 (6)	0.0031 (7)
C9B	0.0530 (8)	0.0559 (9)	0.0571 (9)	0.0014 (7)	0.0163 (7)	0.0059 (7)
C10B	0.0731 (11)	0.0684 (11)	0.0661 (11)	0.0056 (9)	0.0283 (9)	0.0067 (9)
C11B	0.0984 (15)	0.0950 (15)	0.1081 (17)	0.0154 (12)	0.0648 (13)	0.0127 (13)
O1A	0.0507 (6)	0.0923 (9)	0.0765 (8)	−0.0053 (6)	0.0195 (6)	−0.0293 (7)
O2A	0.0473 (6)	0.1230 (11)	0.0829 (9)	−0.0019 (7)	0.0299 (6)	−0.0065 (8)
C6A	0.0473 (8)	0.0721 (10)	0.0563 (9)	0.0017 (7)	0.0194 (7)	−0.0026 (8)
C7A	0.0488 (8)	0.0681 (10)	0.0578 (9)	0.0007 (7)	0.0245 (7)	−0.0049 (8)
C8A	0.0536 (8)	0.0649 (9)	0.0525 (8)	0.0105 (7)	0.0201 (7)	0.0031 (7)
C9A	0.0641 (10)	0.0571 (9)	0.0553 (9)	0.0064 (7)	0.0218 (7)	−0.0006 (7)
C10A	0.0771 (12)	0.0684 (11)	0.0646 (10)	0.0135 (9)	0.0222 (9)	−0.0041 (9)
C11A	0.1050 (16)	0.0634 (11)	0.0814 (14)	0.0037 (11)	0.0172 (12)	−0.0159 (10)

### Geometric parameters (Å, °)

**Table:**

N1—C1	1.332 (2)	C9B—C10B	1.315 (2)
N1—C5	1.358 (2)	C9B—H9BA	0.9300
N1—H1N1	0.89 (3)	C10B—C11B	1.487 (3)
N2—C1	1.332 (2)	C10B—H10A	0.9300
N2—H1N2	0.83 (2)	C11B—H11A	0.9600
N2—H2N2	0.86 (2)	C11B—H11B	0.9600
N3—C2	1.365 (2)	C11B—H11C	0.9600
N3—H1N3	0.843 (18)	O1A—C6A	1.2682 (19)
N3—H2N3	0.92 (2)	O1A—H1A	0.8200
C1—C2	1.423 (2)	O2A—C6A	1.2341 (18)
C2—C3	1.370 (2)	C6A—C7A	1.481 (2)
C3—C4	1.399 (3)	C7A—C8A	1.312 (2)
C3—H3A	0.9300	C7A—H7AA	0.9300
C4—C5	1.339 (3)	C8A—C9A	1.440 (2)
C4—H4A	0.9300	C8A—H8AA	0.9300
C5—H5A	0.9300	C9A—C10A	1.318 (2)
O1B—C6B	1.310 (2)	C9A—H9AA	0.9300
O2B—C6B	1.2192 (17)	C10A—C11A	1.475 (3)
C6B—C7B	1.474 (2)	C10A—H10B	0.9300
C7B—C8B	1.319 (2)	C11A—H11G	0.9600
C7B—H7BA	0.9300	C11A—H11D	0.9600
C8B—C9B	1.445 (2)	C11A—H11E	0.9600
C8B—H8BA	0.9300		
			
C1—N1—C5	123.90 (16)	C10B—C9B—H9BA	117.5
C1—N1—H1N1	119.1 (16)	C8B—C9B—H9BA	117.5
C5—N1—H1N1	117.0 (16)	C9B—C10B—C11B	125.59 (18)
C1—N2—H1N2	114.2 (15)	C9B—C10B—H10A	117.2
C1—N2—H2N2	120.9 (16)	C11B—C10B—H10A	117.2
H1N2—N2—H2N2	124 (2)	C10B—C11B—H11A	109.5
C2—N3—H1N3	123.5 (12)	C10B—C11B—H11B	109.5
C2—N3—H2N3	111.8 (14)	H11A—C11B—H11B	109.5
H1N3—N3—H2N3	119.6 (19)	C10B—C11B—H11C	109.5
N2—C1—N1	119.33 (16)	H11A—C11B—H11C	109.5
N2—C1—C2	122.29 (17)	H11B—C11B—H11C	109.5
N1—C1—C2	118.38 (15)	C6A—O1A—H1A	109.5
N3—C2—C3	123.83 (15)	O2A—C6A—O1A	121.53 (16)
N3—C2—C1	118.61 (14)	O2A—C6A—C7A	121.69 (15)
C3—C2—C1	117.49 (15)	O1A—C6A—C7A	116.77 (13)
C2—C3—C4	121.61 (16)	C8A—C7A—C6A	124.78 (14)
C2—C3—H3A	119.2	C8A—C7A—H7AA	117.6
C4—C3—H3A	119.2	C6A—C7A—H7AA	117.6
C5—C4—C3	119.07 (17)	C7A—C8A—C9A	125.68 (15)
C5—C4—H4A	120.5	C7A—C8A—H8AA	117.2
C3—C4—H4A	120.5	C9A—C8A—H8AA	117.2
C4—C5—N1	119.52 (19)	C10A—C9A—C8A	125.81 (17)
C4—C5—H5A	120.2	C10A—C9A—H9AA	117.1
N1—C5—H5A	120.2	C8A—C9A—H9AA	117.1
O2B—C6B—O1B	122.84 (14)	C9A—C10A—C11A	127.25 (19)
O2B—C6B—C7B	122.85 (15)	C9A—C10A—H10B	116.4
O1B—C6B—C7B	114.30 (13)	C11A—C10A—H10B	116.4
C8B—C7B—C6B	123.09 (14)	C10A—C11A—H11G	109.5
C8B—C7B—H7BA	118.5	C10A—C11A—H11D	109.5
C6B—C7B—H7BA	118.5	H11G—C11A—H11D	109.5
C7B—C8B—C9B	125.75 (14)	C10A—C11A—H11E	109.5
C7B—C8B—H8BA	117.1	H11G—C11A—H11E	109.5
C9B—C8B—H8BA	117.1	H11D—C11A—H11E	109.5
C10B—C9B—C8B	125.09 (16)		
			
C5—N1—C1—N2	−179.49 (19)	O2B—C6B—C7B—C8B	2.3 (2)
C5—N1—C1—C2	1.2 (3)	O1B—C6B—C7B—C8B	−177.53 (15)
N2—C1—C2—N3	−4.5 (3)	C6B—C7B—C8B—C9B	177.96 (14)
N1—C1—C2—N3	174.80 (15)	C7B—C8B—C9B—C10B	−179.26 (17)
N2—C1—C2—C3	178.37 (17)	C8B—C9B—C10B—C11B	179.96 (18)
N1—C1—C2—C3	−2.3 (2)	O2A—C6A—C7A—C8A	−0.6 (3)
N3—C2—C3—C4	−174.93 (17)	O1A—C6A—C7A—C8A	178.42 (16)
C1—C2—C3—C4	2.0 (2)	C6A—C7A—C8A—C9A	−177.50 (15)
C2—C3—C4—C5	−0.5 (3)	C7A—C8A—C9A—C10A	−176.71 (18)
C3—C4—C5—N1	−0.8 (3)	C8A—C9A—C10A—C11A	179.34 (18)
C1—N1—C5—C4	0.4 (3)		

### Hydrogen-bond geometry (Å, °)

**Table:**

*D*—H···*A*	*D*—H	H···*A*	*D*···*A*	*D*—H···*A*
O1A—H1A···O1B	0.82	1.79	2.5252 (19)	148
N1—H1N1···O1A^i^	0.89 (3)	2.06 (3)	2.887 (2)	153 (3)
N1—H1N1···O2A^i^	0.89 (3)	2.31 (3)	3.094 (2)	147 (2)
N2—H1N2···O1A^i^	0.83 (2)	2.30 (2)	3.054 (2)	152 (2)
N2—H1N2···O2B^i^	0.83 (2)	2.59 (3)	3.136 (2)	125 (2)
N2—H2N2···O2A	0.86 (2)	2.00 (3)	2.863 (2)	177 (2)
N3—H1N3···O2A	0.84 (2)	2.19 (2)	3.010 (2)	166.1 (16)
N3—H2N3···O2B^ii^	0.92 (3)	2.09 (3)	3.002 (2)	170 (2)

Symmetry codes: (i) *x*, −*y*+1/2, *z*−1/2; (ii) −*x*+1, −*y*, −*z*+1.

## References

[bb1] Banerjee, S. & Murugavel, R. (2004). *Cryst. Growth Des.* **4**, 545–522.

[bb2] Bernstein, J., Davis, R. E., Shimoni, L. & Chang, N.-L. (1995). *Angew. Chem. Int. Ed. Engl.* **34**, 1555–1573.

[bb3] Bruker (2009). *APEX2*, *SAINT* and *SADABS* Bruker AXS Inc., Madison, Wisconsin, USA.

[bb4] Cosier, J. & Glazer, A. M. (1986). *J. Appl. Cryst.* **19**, 105–107.

[bb5] Hemamalini, M. & Fun, H.-K. (2010). *Acta Cryst.* E**66**, o2369. 10.1107/S1600536810032617 PMC3007997 21588709

[bb6] Lautie, A. & Belabbes, Y. (1996). *Spectrochim. Acta Part A*, **52**, 1903–1914.

[bb7] Leung, M.-K., Mandal, A.-B., Wang, C.-C., Peng, S.-M., Lu, H.-F. & Chou, P.-T. (2002). *J. Am. Chem. Soc.* **124**, 4287–4297. 10.1021/ja011679p 11960458

[bb8] Peng, S.-H., Wang, C.-C., Lo, W.-C. & Peng, S.-M. (2001). *J. Chin. Chem. Soc.* **48**, 987–996.

[bb9] Sheldrick, G. M. (2008). *Acta Cryst.* A**64**, 112–122. 10.1107/S0108767307043930 18156677

[bb10] Spek, A. L. (2009). *Acta Cryst.* D**65**, 148–155. 10.1107/S090744490804362X PMC2631630 19171970

